# Replicates, Read Numbers, and Other Important Experimental Design Considerations for Microbial RNA-seq Identified Using *Bacillus thuringiensis* Datasets

**DOI:** 10.3389/fmicb.2016.00794

**Published:** 2016-05-31

**Authors:** Punita Manga, Dawn M. Klingeman, Tse-Yuan S. Lu, Tonia L. Mehlhorn, Dale A. Pelletier, Loren J. Hauser, Charlotte M. Wilson, Steven D. Brown

**Affiliations:** ^1^Graduate School of Genome Science and Technology, University of TennesseeKnoxville, TN, USA; ^2^BioEnergy Science Center, Oak Ridge National LaboratoryOak Ridge, TN, USA; ^3^Biosciences Division, Oak Ridge National LaboratoryOak Ridge, TN, USA; ^4^Environmental Sciences Division, Oak Ridge National LaboratoryOak Ridge, TN, USA

**Keywords:** replicates, DESeq2, negative binomial, Illumina, normalization, coverage

## Abstract

RNA-seq is being used increasingly for gene expression studies and it is revolutionizing the fields of genomics and transcriptomics. However, the field of RNA-seq analysis is still evolving. Therefore, we specifically designed this study to contain large numbers of reads and four biological replicates per condition so we could alter these parameters and assess their impact on differential expression results. *Bacillus thuringiensis* strains ATCC10792 and CT43 were grown in two Luria broth medium lots on four dates and transcriptomics data were generated using one lane of sequence output from an Illumina HiSeq2000 instrument for each of the 32 samples, which were then analyzed using DESeq2. Genome coverages across samples ranged from 87 to 465X with medium lots and culture dates identified as major variation sources. Significantly differentially expressed genes (5% FDR, two-fold change) were detected for cultures grown using different medium lots and between different dates. The highly differentially expressed iron acquisition and metabolism genes, were a likely consequence of differing amounts of iron in the two media lots. Indeed, in this study RNA-seq was a tool for predictive biology since we hypothesized and confirmed the two LB medium lots had different iron contents (~two-fold difference). This study shows that the noise in data can be controlled and minimized with appropriate experimental design and by having the appropriate number of replicates and reads for the system being studied. We outline parameters for an efficient and cost effective microbial transcriptomics study.

## Introduction

Ever decreasing next-generation sequencing (NGS) costs, continued technical and analytical advances, along with diverse applications have made RNA-sequencing (RNA-seq) an ever increasing choice for transcriptome studies (Croucher and Thomson, 2010; Marguerat and Bahler, 2010; Williams et al., 2014). RNA-seq applications include differential gene expression studies, the detection of strand specific expression or transcript fusions, determination of alternative splicing isoforms, identification of specific SNP's and their locations, long and small RNAs, genome guided, and *de novo* transcript assemblies and start sites analyses (Martin and Wang, 2011; McGettigan, 2013; Mutz et al., 2013). It also enables detection of weakly expressed genes and does not have to be limited by previously sequenced genome knowledge (Marguerat and Bahler, 2010).

Various NGS platforms, assembly and statistical tools can be used to generate RNA-seq datasets, but the overall methodology across platforms is similar (Williams et al., 2014). While direct RNA sequencing is possible (Ozsolak et al., 2009), for the majority of expression studies RNA is isolated from cells and usually undergoes rRNA depletion or poly(A) enrichment. The transcript enriched material is then used as template material to generate complementary DNA (cDNA) libraries via a reverse transcription enzymatic reaction, which represents the transcripts within each sample. Library creation may include the addition of barcodes/adaptors so samples from multiple conditions can be pooled, run together, and then data attributed appropriately. In the case of indirect RNA-seq methods an amplification step is required. Sequence data in the form of raw reads are quality filtered/trimmed, most often aligned to a reference genome, then the number of reads mapped to individual genes in the reference genome are counted and then further used to estimate differential gene expression using a range of statistical methods (Auer and Doerge, 2010; Marguerat and Bahler, 2010; Oshlack et al., 2010).

While RNA-seq has a number of advantages over DNA microarrays, it is still a developing technology that faces a number of challenges (Wang et al., 2009; Ozsolak and Milos, 2011; Mutz et al., 2013; Peixoto et al., 2015). Variation, errors, and biases may be introduced in any of the multiple steps used to generate and analyze the datasets (Pinto et al., 2011). Technical and biological factors that contribute to variation, errors, and biases include experimental design, RNA extraction procedures, sample handling, differences in amount of starting RNA, library preparation steps such as PCR amplification, sample storage, GC content, and read number differences (Fang and Cui, 2011; Peixoto et al., 2015). A number of different of normalization methods have been developed for NGS data to remove unwanted variance (Robinson and Oshlack, 2010; Dillies et al., 2013). Normalization methods include examples such Total Count (TC), Upper Quartile (UQ), Reads Per Million base pairs (RPM), Reads Per Kilobase per Million base pairs (RPKM), Trimmed Mean of M-values (TMM), Kernel Density Mean of M-component (KDMM), and analysis packages like DESeq and edgeR have inbuilt normalization algorithms (Anders and Huber, 2010; Robinson and Oshlack, 2010; Anders et al., 2013; Dillies et al., 2013; Love et al., 2014). There is no clear consensus on which normalization is the best suited for RNA-seq data. Although, studies that have compared some of these methods to one another show that UQ, TMM, and DESeq normalization result in similar qualitative characteristics of the normalized dataset and differential expression analysis (Dillies et al., 2013; Soneson and Delorenzi, 2013). Recent studies have shown that RNA-seq data often fits well to a negative binomial distribution (Miller et al., 2011; Li and Tibshirani, 2013; Gierlinski et al., 2015; Mi et al., 2015) and this method is being more widely adopted. While a well-designed experiment and normalization are important, they may be insufficient if there is large unknown variance (Peixoto et al., 2015). A recent study analyzed RNA-seq data from 48 samples obtained from seven Illumina HiSeq 2000 lanes and concluded that “bad” replicates risk skewing data interpretation and that increasing biological replicates beyond the typical two or three is beneficial (Gierlinski et al., 2015).

Other important considerations for an RNA-seq study include the choice of quality trimming/filtering tools, mapping algorithm, statistical test, required number of reads, or genome coverage, number of biological replicates and cost (Pinto et al., 2011; Liu et al., 2014; Peixoto et al., 2015). NGS technologies generate large datasets that may be computationally challenging for smaller laboratories to store, retrieve, and analyze. Thus, there is a demand for bioinformatic tools that are proficient in data handling i.e., are fast and have reduced error rates, have a broad consensus and are easy to use (Wang et al., 2009; Auer and Doerge, 2010; Fonseca et al., 2012; Sims et al., 2014). Cost is an essential factor for most laboratories, which is directly related to the number of reads generated per sample and the number of replicates used. Thus, it is important to establish an acceptable trade-off between number of reads and replicates for an efficient, powerful, yet cost effective experiment (Liu et al., 2014).

The aim of the present study was to better understand the required number of reads and replicate numbers for statistically confident results in the context of a typical experimental laboratory. Transcriptomic profiles were generated for two closely related *Bacillus thuringiensis* strains (serovar berliner ATCC10792 and CT43) under similar experimental conditions and since the outcomes for each strain were similar to one another we mainly present ATCC10792 analyses and analyses for strain CT43 are shown as Supplementary Material. *B. thuringiensis* is a Gram-positive, spore and Cry toxin producing bacterium (Joung and Cote, 2001) that has been applied for biocontrol of different insects (Baxter et al., 2011; Bravo et al., 2011; Gassmann et al., 2014) and a number of genome sequences are available for study (He et al., 2011; Johnson et al., 2015). The ATCC 10792 genome (NCBI accession NZ_CM000753) is 6,260,142 bp, was recently reannotated with 6330 genes and 13 copies for the 5S, 16S, and 23S rRNA genes predicted. The data from this well-replicated study with 32 samples, each from one Illumina HiSeq 2000 lane, generated a large number of reads per sample, and significantly differentially expressed genes were detected using DESeq2 (Love et al., 2014). Differentially expressed (DE) genes were validated by Real Time quantitative RT-qPCR. This study provides insights into sample and read numbers required to derive biologically meaningful results and will be useful to others looking to develop or assess different bioinformatics and/or statistical approaches for RNA-seq studies.

## Materials and methods

### Organism growth and sampling

**Figure 1:**
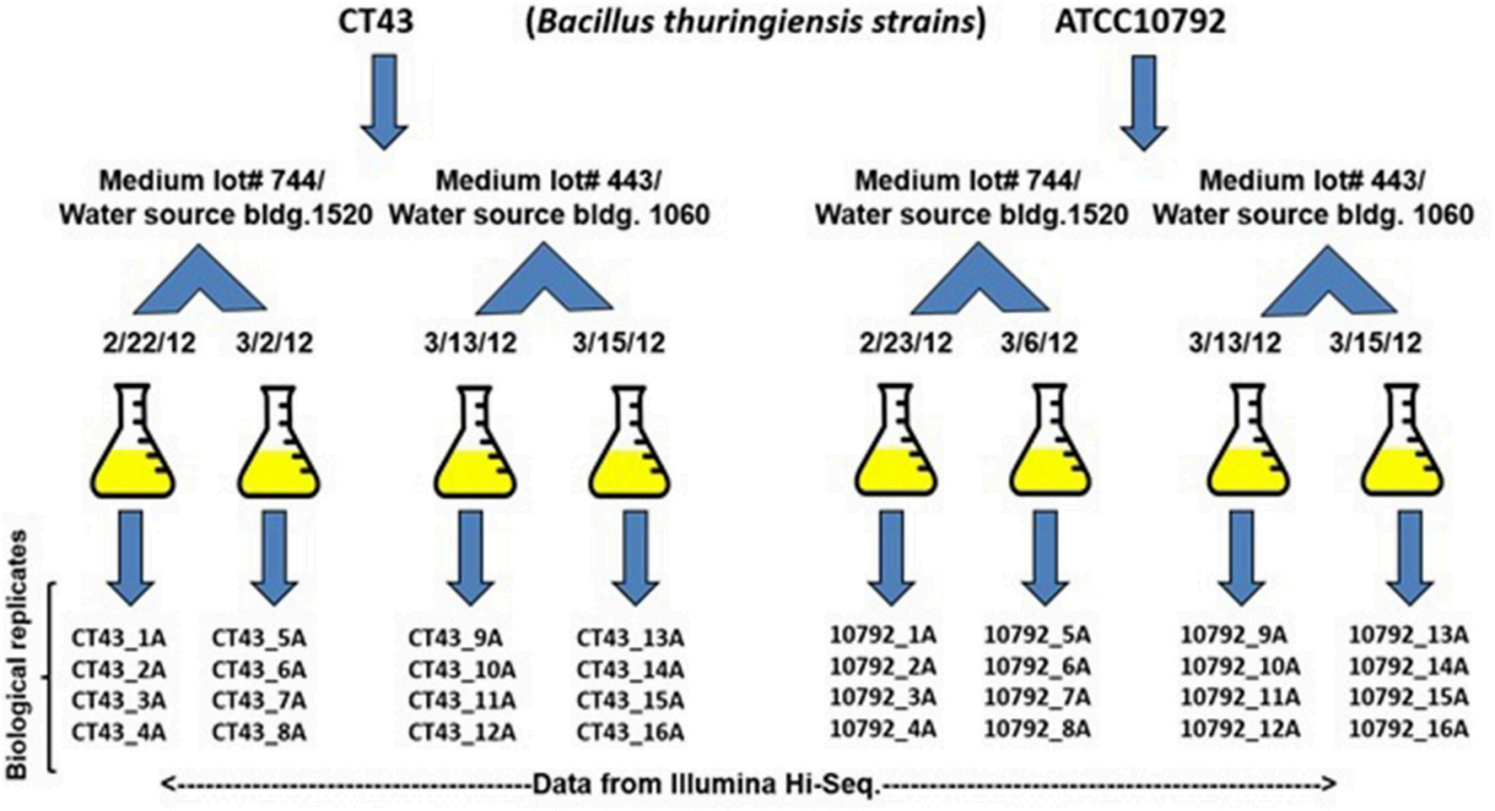
**Sampling and major sources of variation**. Strains CT43 and ATCC10792 grown in two medium lots #1091744 and 7220443 in water taken from building 1520 and 1610. Bacteria were cultured on four different dates and four biological replicates were grown to mid-log phase for each date, harvested and then RNA-seq data were generated using an Illumina Hiseq 2000 instrument.

*Bacillus thuringiensis* serovar *berliner* strain ATCC 10792 and *Bacillus thuringiensis* serovar *chinensis* strain CT-43 were obtained from the *Bacillus* Genetic Stock Center (www.bgsc.org) and have Average Nucleotide Identity (ANI)-values of ≥99.63% in reciprocal genome analyses based on BLAST (ANib). Each strain was plated on Luria Bertani (LB) medium and cultured at 30°C. Single colonies were used to inoculate 5 mL LB starter cultures, which were grown at 30°C with shaking at 200 rpm (New Brunswick Scientific, Innova 4430) overnight. For RNA-seq experiments, 1 mL aliquots of overnight cultures were used to inoculate 500 mL baffled flasks containing 200 mL of LB medium. Cultures were grown for 3 h at 30°C with shaking at 200 rpm and harvested at approximately mid-log phase (OD_600_, ~0.42). To harvest cells for RNA extraction, 40 mL culture aliquots were collected by rapid centrifugation (Sorvall, Evolution RC) at 7649 × g at 4°C for 5 min. Cell pellets were frozen in liquid nitrogen for 10 min and then stored at −80°C. All cultures were grown and harvested under similar conditions. A total of 16 samples were collected per strain, with four biological replicates for each strain, collected on four different dates and using media from two different LB broth lots (lot #1091744 and 7220443) using water from two different buildings to generate 32 samples for RNA-seq analysis (Figure 1, A summary for all supplementary material is provided Data Sheet 1. Data Sheet 2 contains growth and RNA-seq analysis). Difco Lennox LB medium was used in this study (Becton, Dickinson and Company, Franklin Lakes, NJ, USA).

### RNA extraction and cDNA library preparation

High quality RNA (Bioanalyzer RNA integrity numbers (RINs) >8.5) was isolated from strain CT43 and strain ATCC 10792 using the TRIzol reagent (Invitrogen, Carlsbad, CA, USA) combined with a bead beating step, essentially as described previously (Wilson et al., 2013). Briefly, cell pellets from each sample were resuspended in TRIzol reagent, then TRIzol/cell mixtures were added to tubes containing 800 mg of 0.1 mm glass beads (Biospec Products Inc., Bartlesville, OK, USA) and cells were lysed by bead beating on a Precellys 24 high-throughput tissue homogenizer (Bertin Technologies, Montigny-le-Bretonneux, France) with the following settings; 3 × 20 s at 6500 rpm. Chloroform was added post-lysis, mixed by vortexing, and the mixture was centrifuged at 20,817 × g (Centrifuge 5417R, Eppendorf) for 15 min at 4°C. The aqueous phase was collected and mixed with ethanol and purified using the RNeasy Mini kit (Qiagen, Waltham, MA, USA) in accordance with the manufacturer's instructions and using the optional on column DNaseI treatment. The quantity and quality of RNA was assessed using a NanoDrop ND-1000 spectrophotometer (NanoDrop Technologies, Wilmington, DE) and Agilent Bioanalyzer (Agilent, Santa Clara, CA, USA). Ribosomal RNA was depleted from the samples using a Ribo-Zero rRNA removal kit for Gram-Positive bacteria (Epicentre, Madison, WI, USA) and cDNA libraries were prepared and barcoded using a ScriptSeq v2 RNA-seq Library preparation kit. The final libraries were quantified with a Qubit double stranded broad range assay kit and fluorometer (Invitrogen) and quality assessed using a Bioanalyzer (Agilent). Samples were diluted according to manufacturer's recommendations using the Illumina dilution calculator, and sequence data was generated via two runs using an Illumina HiSeq 2000 instrument with SR50 sequencing kits (50 bp single end reads) and using phiX control DNA (Illumina, Inc., San Diego, CA, USA), as previously described (Wilson et al., 2013).

### Data analysis

#### Mapping, clustering, and quality control

Raw reads were imported into the CLC Genomics Workbench 7.0.4 (CLCBio, a Qiagen company) and were filtered and trimmed based on quality assessments. Sequence reads < 20 nucleotides were discarded. A modified-Mott trimming algorithm, which incorporates quality scores on a Phred scale, was used for quality trimming in CLCBio with the quality trimming parameter set to 0.02. Trimmed and filtered reads were then aligned to their respective reference genomes, using the default “prokaryote genomes” and unique reads settings. Raw data variance was observed by Principle Component Analysis (PCA) and by cluster analysis using JMPGenomics 6.0 (SAS Institute, Cary, NC, USA).

#### Data access

The genomes used in this study have been described (He et al., 2011; Johnson et al., 2015) and are available from the NCBI GenBank database under accession numbers for NZ_CM000753 and NC_017208 for strains ATCC10792 and CT43, respectively. RNA-seq data have been deposited in NCBI Gene Expression Omnibus (GEO) database under accession number GSE71189 and raw sequence data deposited at the NCBI Sequence Read Archive (SRA) under accession number SRP041628.

### Differential gene expression analysis: DESeq2

Uniquely mapped reads were incorporated into a tabular format (Data Sheet 3) and analyzed using the DESeq2 differential expression analysis pipeline (Love et al., 2014). Differentially expressed (DE) genes were identified based on comparisons between medium lots and culture dates for each strain using a 5% False Discovery Rate (FDR) and a two-fold expression difference to detect significantly DE genes (Data Sheets 4–6).

### RT-qPCR validation of RNA-seq results

RNA-seq data for the differentially expressed genes was validated using real-time quantitative PCR (RT-qPCR) as previously described (Wilson et al., 2013). Six *B. thuringiensis* strain ATCC10792 genes that represented a range of differential expression values from RNA-seq data were chosen for validation. Primers used to validate medium and the date effects are listed in Data Sheet 7.

### Determination of iron content in media and water

Iron content for the LB medium and for the water sources from the two different building sources were quantified by elemental analysis using a Perkin Elmer ELAN 6100 Inductively Coupled Plasma-Mass Spectrometer (ICP-MS), as previously described (Zhang et al., 2010). Water source and media lot pH were measured using colorpHast pH-indicator strips (EMD Millipore, Billerica, MA, USA).

### Alteration of sequence read and biological replicate numbers

To observe the effect of using fewer biological replicates and lower sequence read numbers on differential gene expression detection, data available from all biological replicates per strain within each condition (i.e., same medium lot and date of culture) were grouped in sets with replicates for another condition (Figure 1). The number of biological replicates varied from two to four and the number of differentially expressed genes were determined using DESeq2 (Data Sheet 8). For example, a set of two replicates 1A/2A were grouped with 9A/10A for differential expression estimation. When analyzing the effect that the total number of reads per sample had on the number of differentially expressed genes, the original number of reads obtained for each sample with genome coverage ranges from 87 to 465x was considered as 100% reads (Supplementary File 1. Subsets with randomly reduced reads of 75, 50, 25, 10, and 5% of the original number of reads were generated using the “sample reads” option in the genome finishing module of CLC Genomics Workbench 7.0.4 (CLCBio). Each subset was remapped with the respective reference genome prior to performing differential gene expression analysis via DESeq2 (Data Sheet 9).

## Results

### RNA-seq experiments

**Figure 2:**
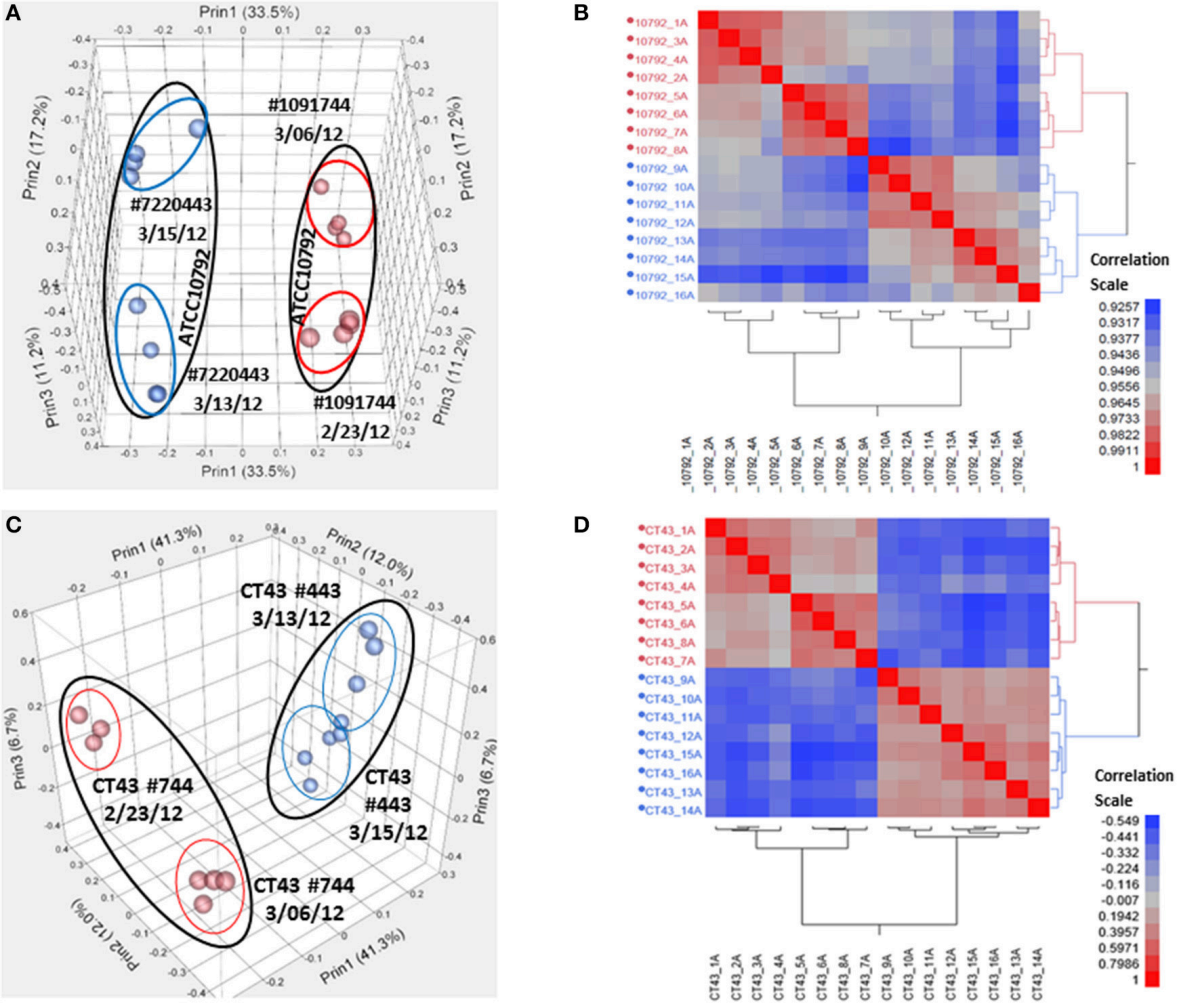
**Variation analysis of raw read count data for strain ATCC10792 and strain CT43. (A)** Principal Component Analysis (PCA) for ATCC10792 using a Pearson correlation coefficient and colored by media, **(B)** Hierarchical cluster analysis of the same data for strain ATCC10792, **(C)** PCA for CT43 and **(D)** CT43 cluster analysis.

Samples were harvested for all cultures during mid-exponential growth. The average culture turbidity, as measured by optical density at OD_600*nm*_, was 0.422 ± 0.04 (range 0.384–0.504) for strain ATCC10792 and 0.415 ± 0.05 for CT43 at the time of sample collection (Table 1 and Supplementary File 1). For each sample 15–30 M raw reads were generated and the resulting genome coverages were between 87 and 465X post-quality filtering and trimming. Post-trimming and mapping results for strain ATCC10792 is provided in Table 1 and similar results were obtained for strain CT43 (Supplementary File 1). The ribosomal RNA depletion strategy worked well and similarly for both strains as indicated by an analysis showing that on average for both strains only 0.07% of trimmed, mapped reads aligned to the 5S, 16S, and 23S rRNA genes (S.D. ± 0.05 and 0.06 for ATCC 10792 and CT43, respectively). For both strains, medium lot and culture date were identified as important variance sources during Principal Component Analysis (PCA) and cluster analysis of raw data (Figure 2). Variation across biological replicates was low with the linear (Pearson) correlation values within like replicates for both ATCC10792 and CT43 ranging from between 0.95 and 0.99 (Data Sheet 7).

**Table 1:** **Summary of trimmed and mapped reads for strain ATCC10792**.

**ATCC10792 (Ref. genome size = 6260142)**	**Sample OD_600_**	**Total no. of reads (trimmed)**	^*^Genome coverage	**Total mapped reads to CDSs**	**Unique reads to CDSs**
1A	0.394	26,986,606	202x	19,762,858	19,708,701
2A	0.398	11,787,714	87x	7,579,068	7,534,508
3A	0.384	27,315,600	203x	19,174,331	19,098,676
4A	0.404	53,643,496	400x	37,447,561	37,326,511
5A	0.398	51,109,286	380x	37,229,691	37,112,202
6A	0.420	57,636,652	430x	41,566,889	41,466,848
7A	0.406	49,915,906	370x	34,855,294	34,747,534
8A	0.452	52,689,519	392x	37,104,818	36,999,659
9A	0.384	20,291,318	160x	9,640,962	9,590,311
10A	0.386	13,356,476	105x	6,185,482	6,154,691
11A	0.398	22,487,034	177x	9,762,915	9,698,237
12A	0.392	25,052,676	197x	10,408,386	10,361,349
13A	0.482	21,857,603	172x	9,475,191	9,444,912
14A	0.476	27,286,565	215x	10,871,957	10,828,524
15A	0.504	26,104,043	206x	9,558,505	9,484,938
16A	0.468	30,818,722	243x	21,037,052	20,987,988

* *See Data Sheet 2 for calculation details*.

### Differential gene expression analysis using DESeq2: medium lot and date effect

As variation based on medium lots and culture dates was detected, differential expression analysis was conducted to examine the effect of different media, and culture dates on transcriptomic profiles. When all replicates per strain and 100% reads were applied to the analysis, 735 and 1086 genes (5% False Discovery Rate (FDR) and two-fold change) were observed to be differentially expressed between medium lots (#1091744 vs. #7220443) for strains ATCC10792 and CT43, respectively. A complete list of altered gene expression based on medium lot difference is provided in Supplementary File 3 for ATCC10792 and CT43. In response to the different medium lots, genes related to iron acquisition and metabolism were consistently differentially expressed for both strains. A summary of iron related genes that passed both 5% FDR and two-fold expression difference significance thresholds is shown (Table 2, ATCC10792; Data Sheet 4 for CT43). Based on the differential expression results, it was hypothesized that iron had become limiting for cultures grown in medium from lot 1091744 compared to cells grown in medium prepared from the other lot. An elemental analysis of sterile media prepared from different lots revealed that higher amounts of total iron were indeed present in medium lot #7220443 compared to medium lot #1091744 (Table 3). Both media were prepared at pH ~ 7.0.

**Table 2:** **Genes related to iron acquisition and metabolism differentially expressed in strain ATCC10792 grown in medium lot #1091744 over #7220443**.

**Differentially expressed Iron genes (medium lot #744 vs. #443)**			
**Locus_tag**	**Product**	**log2 fold change**	**Padj (FDR** = **5%)**
BTHUR0008_RS01670	Iron ABC transporter permease	2.67	<0.001
BTHUR0008_RS01675	Ferrichrome ABC transporter permease	2.70	<0.001
BTHUR0008_RS01680	ABC transporter substrate-binding protein	2.94	<0.001
BTHUR0008_RS01685	Ferredoxin–NADP reductase	2.56	<0.001
BTHUR0008_RS02820	Iron-enterobactin transporter ATP-binding protein	1.30	<0.001
BTHUR0008_RS02825	Iron ABC transporter permease	1.45	<0.001
BTHUR0008_RS02835	Iron siderophore-binding protein	1.23	<0.001
BTHUR0008_RS03465	Iron transporter FeoA	−1.23	<0.001
BTHUR0008_RS06975	Ferredoxin	−1.04	<0.001
BTHUR0008_RS10095	Fe-S oxidoreductase	−1.43	<0.001
BTHUR0008_RS10345	Iron(III) dicitrate-binding protein	1.96	<0.001
BTHUR0008_RS15775	Ferrichrome ABC transporter permease	2.50	<0.001
BTHUR0008_RS15780	Iron ABC transporter permease	2.34	<0.001
BTHUR0008_RS15785	Iron-hydroxamate ABC transporter substrate-binding protein	2.51	<0.001
BTHUR0008_RS17445	Iron-uptake system-binding protein	3.45	<0.001
BTHUR0008_RS17450	Ferrichrome ABC transporter permease	2.88	<0.001
BTHUR0008_RS17455	Iron ABC transporter permease	3.72	<0.001
BTHUR0008_RS17460	ABC transporter ATP-binding protein	3.40	<0.001
BTHUR0008_RS17465	IroE protein	2.50	<0.001
BTHUR0008_RS20850	Iron ABC transporter ATP-binding protein	3.70	<0.001
BTHUR0008_RS20855	Iron ABC transporter permease	3.24	<0.001
BTHUR0008_RS20860	Iron-hydroxamate ABC transporter substrate-binding protein	4.02	<0.001
BTHUR0008_RS21120	Ferrichrome ABC transporter substrate-binding protein	2.70	<0.001
BTHUR0008_RS21675	Ferrichrome ABC transporter substrate-binding protein	1.60	<0.001
BTHUR0008_RS21745	Heme-degrading monooxygenase IsdG	2.41	<0.001
BTHUR0008_RS21760	ABC transporter permease	1.40	<0.001
BTHUR0008_RS21765	Heme ABC transporter substrate-binding protein	2.31	<0.001
BTHUR0008_RS23110	Iron transporter FeoA	1.13	<0.001
BTHUR0008_RS23575	Ferritin	−1.60	<0.001
BTHUR0008_RS25020	Iron ABC transporter substrate-binding protein	2.17	<0.001
BTHUR0008_RS25025	Iron ABC transporter permease	1.70	<0.001
BTHUR0008_RS25030	Iron ABC transporter permease	1.31	<0.001
BTHUR0008_RS25035	Iron ABC transporter ATP-binding protein	1.10	<0.001
BTHUR0008_RS25920	Ferrichrome ABC transporter permease	1.74	<0.001
BTHUR0008_RS25930	Iron-dicitrate ABC transporter ATP-binding protein	1.30	<0.001
BTHUR0008_RS25935	Ferrichrome ABC transporter substrate-binding protein	2.72	<0.001

**Table 3:** **Elemental analysis of the two media lots and water sources**.

**Media sample**	**Total Fe (ppm)**	**Fe^2+^ (ppm)**
Lot #1091744 1520	0.15 ± 0.01	0.02 ± 0.02
Lot #7220443 1060	0.30 ± 0.01	0.07 ± 0.02
**WATER SOURCE (BUILDING)**		
1520	0.01 ± 0.01	
1060	0.01 ± 0.00	

When analyzing the data based on different culture dates within a particular medium lot (the date effect) for ATCC10792, 403 genes were identified as differentially expressed for cultures in medium lot #1091744 when culture from the date 2/23/12 was compared with 3/6/12. Similarly, for cultures grown in medium lot #7220443 when comparing cultures from dates 3/13/12 and 3/15/12, 458 genes were identified as differentially expressed in ATCC10792 (Supplementary File 4). Similar results were obtained for strain CT43 when differential gene expression analysis was conducted for the culturing date effect within a particular medium lot (Data Sheet 6).

### Real time-quantitative PCR validation (RT-qPCR)

**Figure 3:**
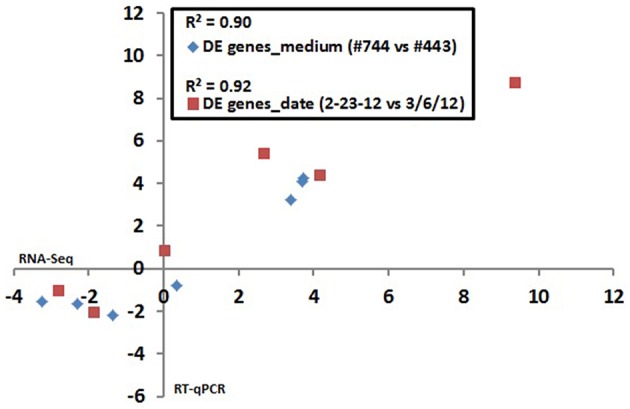
**RNA seq data validation: Correlation between RNA seq and RT qPCR results for differential gene expression in ***Bt*** strain ATCC10792 grown on different medium lots (#744–443) and dates (2/23/12–3/6/12)**. The log_2_ transformed expression ratio values from RNA seq (x-axis) and RT qPCR (y-axis) were plotted against each other and correlation coefficient (*R*^2^)-values were calculated. Seven genes plotted for medium effect: BTHUR0008_RS06920, BTHUR0008_RS03645, BTHUR0008_RS15085, BTHUR0008_RS17455, BTHUR0008_RS20850, BTHUR0008_RS17460 and BTHUR0008_RS19345. Six genes plotted for date effect in samples from medium Lot #744 (2/23/12 vs. 3/6/12): BTHUR0008_RS30620, BTHUR0008_RS19140, BTHUR0008_RS01820, BTHUR0008_RS21040, BTHUR0008_RS26070 and BTHUR0008_RS08955.

Six genes exhibiting a broad range of strain ATCC10792 expression differences for both medium lot and date effect comparisons were selected for confirmation by RT-qPCR. Comparison of DESeq2 estimated expression differences with measurements determined by RT-qPCR showed that the two different data sets had correlation coefficient (*R*^2^)-values of 0.90 and 0.92 for genes chosen for medium lots and culture dates, respectively (Figure 3).

### Effect of reduced number of replicates and reads on differential gene expression detection

In order to investigate the number of reads and replicates required to detect differentially expressed genes with confidence, the number of reads as well as replicates were varied, and the subsequent outcome on differential gene detection was determined. Based on knowledge from the literature as well as a realistic range for number of replicates in any biological study considering time, money and sample availability, here we focused on two to four replicates.

**Figure 4:**
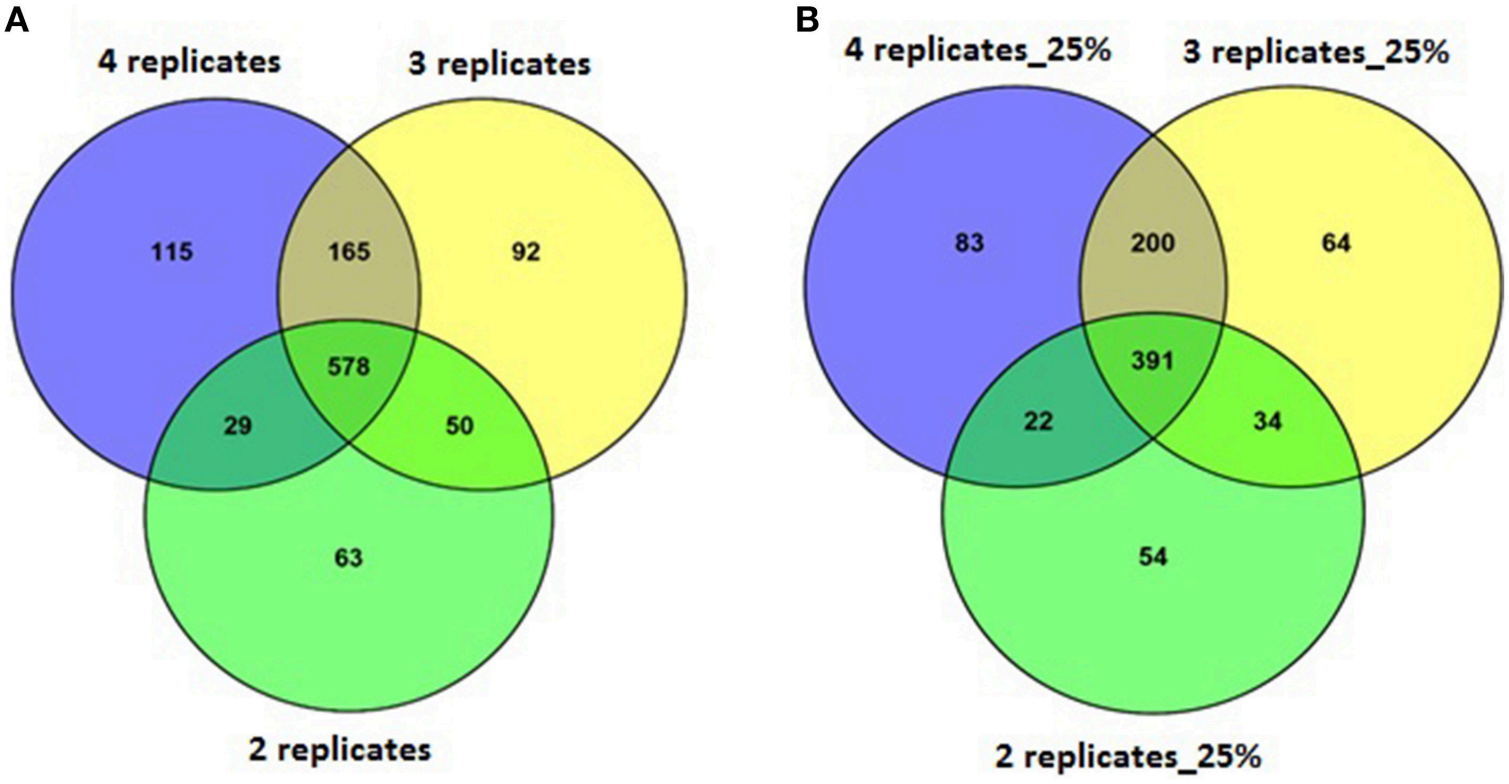
**Venn analysis of DE genes detected with varying replicate numbers. (A)** Venn diagram depicting the effect of 2–4 replicates while maintaining 100% of the reads, on significantly differentially expressed genes and the genes commonly detected within sets of varying replicates for strain ATCC10792. **(B)** Venn diagram for differentially expressed genes detected with 25% reads (~5–10 M) and 2–4 replicates for strain ATCC10792.

**Figure 5:**
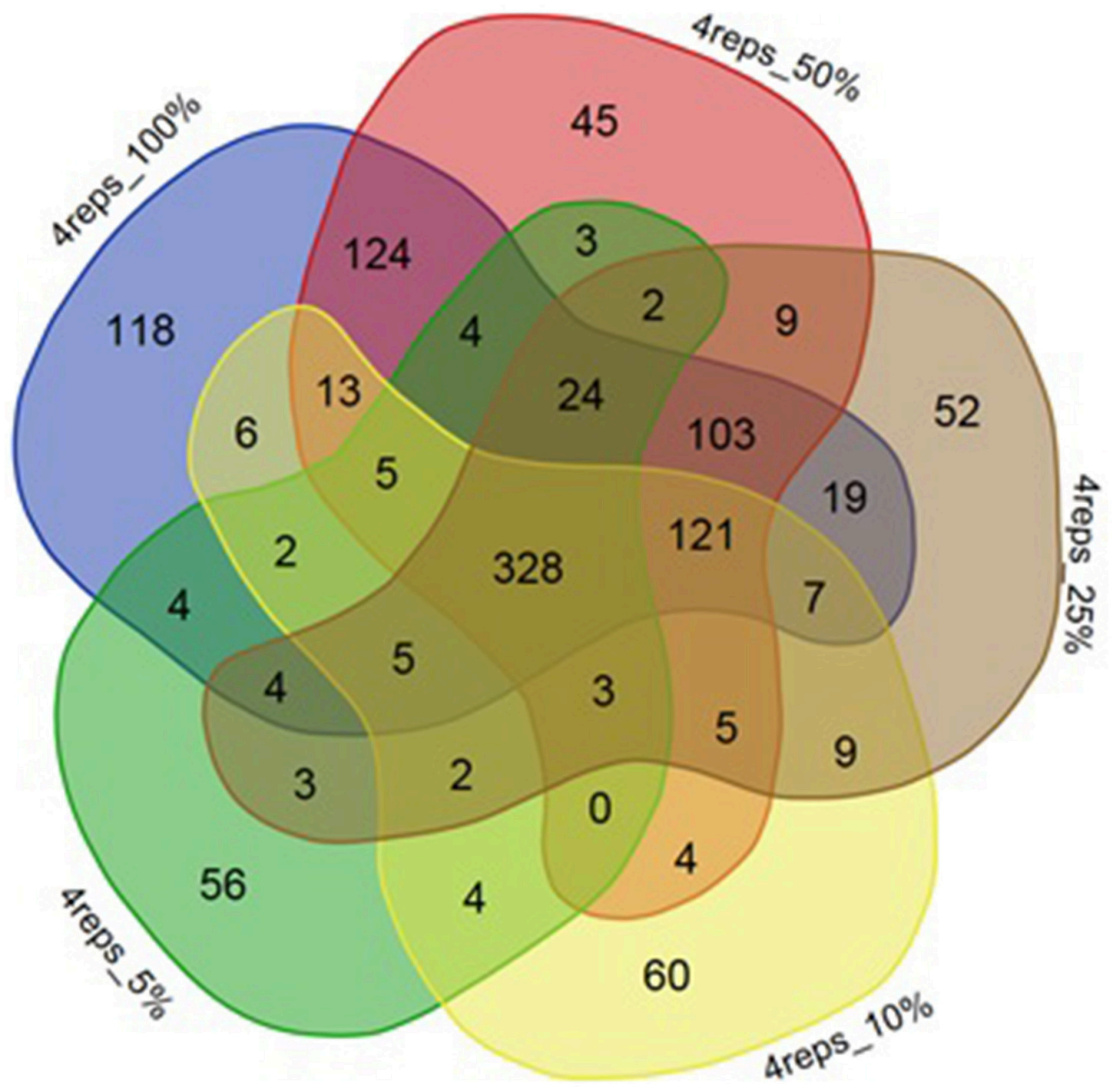
**Venn diagram depicting effect of reducing number of reads on DE gene numbers**. The effect of decreasing of read numbers on significantly differentially expressed genes and the number of genes commonly detected within sets of 100, 50, 25, 10, 5% reads for strain ATCC10792.

A set of four replicates from each medium lot and any one of the two culture dates with 100% of the trimmed reads (~12–58 M) was analyzed (Table 4). A total of 887 genes were detected as significantly differentially expressed upon analyzing the differential gene expression between medium lots for ATCC10792 at thresholds of two-fold differential gene expression and FDR ≤ 0.05. Analyses that included sets of three and two replicates led to the detection of 885 and 720 differentially expressed genes, respectively (Table 4). The significantly differentially expressed genes with three replicates had 743 and two replicates had 607 genes common with the results from the four replicates analysis (Figure 4A). In order to examine how fewer biological replicate numbers affected differential gene expression results for an experiment containing a modest number of reads, the 25% read dataset was selected for further analysis. The 25% read dataset was created by randomly removing 75% of the total reads that had been filtered and trimmed for quality (100%; ~12–58 M reads). The sets of replicates and their reduced (25%) read coverages were: 1A, 2A, 3A, 4A (~3–13 M) vs. 9A, 10A, 11A, 12A (~3–6 M) for analysis with all four replicates; 1A, 3A, 4A (~7–13 M) vs. 9A, 11A, 12A (~5–6 M) for three replicates and 1A, 4A (~7–13 M) vs. 9A, 12A (~5–6 M) two replicates, which gave 696, 689, and 501 significantly differentially expressed genes, respectively (Table 4). There were 591 genes detected with three replicates and 413 genes detected with two replicates that were in common with the set of four replicates when the 25% subset of reads was analyzed for all (Figure 4B). Four out of the seven RT-qPCR validated genes (BTHUR0008_RS03645, BTHUR0008_RS17455, BTHUR0008_RS20850, and BTHUR0008_RS17460) were among the genes considered significant for all conditions in 25% read dataset analysis. Moreover, the same four genes were also considered significant for analyses that included four, three, and two replicates with 100% of the available reads; as well as for analyses that contained 5–100% reads and compared four replicates (Figures 4, 5).

**Table 4:** **Effect of decreasing number of replicates on significantly differentially expressed genes while maintaining 100 and 25% of the reads**.

Number of replicates (ATCC10792)^*^	**Differentially expressed genes (FDR 5%, two-fold)**		**Genes commonly detected with all four reps**	
	**100%Reads**	**25%Reads**	**100%Reads**	**25%Reads**
4 (1A, 2A, 3A, 4A)/(9A, 10A, 11A, 12A)	887	696	100% (887)	100% (696)
3 (1A, 3A, 4A)/(9A, 11A, 12A)	885	689	83.5% (741)	84.9% (591)
2 (1A, 4A)/(9A,12A)	720	501	68.4% (607)	59.3% (413)

* *All combinations of available replicates were tested. Results for replicates with most similar read numbers are shown*.

Selection of an appropriate sequencing depth or genome coverage is a concern in the field, which impacts sensitivity, detection of weakly expressed genes as well as considerations such as cost and replicate numbers (Fang and Cui, 2011; Liu et al., 2014; Williams et al., 2014). The outcome of reducing read numbers on the detection of differentially expressed genes was examined in this study. The initial quality trimmed and filtered reads for strain ATCC10792 (~12–58 M/sample, Supplementary File 1) are referred to as 100% of the reads, which were randomly subsampled to generate input files with 75, 50, 25, 10, and 5% (Supplementary File 7) of the total available reads for a four replicate differential gene expression analysis of cells grown in different media lots. A trend of fewer genes being considered as differentially expressed was observed as fewer reads were incorporated into the analysis (Table 5) and there was a core of 328 DE genes regardless of differing read levels (Figure 5).

**Table 5:** **Effect of decreasing number of reads on significantly differentially expressed genes while maintaining all four replicates**.

**Number of reads (ATCC10792) (%)**	**Differentially expressed genes (FDR 5%, two-fold)**	**Genes commonly detected with 100% reads**
100	887	100% (887)
75	843	95% (803)
50	793	89.4% (722)
25	696	78.5% (611)
10	574	64.7% (487)
5	449	50.6% (376)

Along with the genes that were commonly detected as significantly differentially expressed between datasets of varying number of biological replicates and/or numbers of reads, the genes that were exclusively detected within each of these datasets was also important. Between datasets of four and three replicates using 100% of the reads, 144 genes were exclusively detected as differentially expressed within the four replicates dataset (Figure 4A). When these 144 genes were examined in the dataset consisting of three replicates, it was found that ~60% (87 of 144 genes) were not detected in the differentially expressed gene list as they fell below the two-fold expression threshold. Approximately 24% (34 of 144 genes) dropped below the FDR threshold due to minor deviations from the set limits. For example, one of the genes “BTHUR0008_RS07395” had a difference of 0.04 between the log 2-fold values from four and three replicates and thus fell below the fold-change cut-off with a difference of 0.02; yet another gene “BTHUR0008_RS29930” had an adjusted *p*-value difference of –0.04 between values from four and three replicates and dropped out at the FDR set threshold with a difference of 0.01. The remaining 16% (23 of 144 genes) did not show up in the differentially expressed genes as their *p*-adjusted values were set to “NA” by automatic independent filtering based on low mean normalized counts in DESeq2. Similarly, for datasets of four and two replicates (Figure 4A), out of the 280 genes that were exclusively detected as differentially expressed with four replicates, almost half (139/280) of the genes fell below the two-fold threshold, ~11% (32/280) dropped below FDR cut-off and ~40% (109/280) were left out due to independent filtering in DESeq2. Those genes that were exclusive to sets of two and three replicates but were not detected as differentially expressed with four replicates (Figure 4A), were also seen to be left out because of the same reasons as mentioned above, which led to their not meeting the significant differential expression criteria. A similar trend was also observed for the genes exclusively detected to datasets of four, three and two replicates with 25% of the reads (Figure 4B) as well as for such genes between datasets maintaining all four replicates but reads varying from 5 to 100% (Figure 5).

## Discussion

In this study, we analyzed large transcriptomic datasets from *B. thuringiensis* ATCC10792 and CT43 using negative binomial distribution in DESeq2 for the assessment of differential gene expression. Analysis was performed in different combinations of the data sets to better understand the major challenges of experimental design, variation, required number of replicates and adequate sequencing depth associated with RNA-seq data analysis. Based on our results, as well as previously reported comparisons, we outline important considerations and provide design recommendations for cost-effective RNA-seq results with sufficient statistical power. Improving NGS technologies and instrumentation has led to reproducible results with little technical variation (Marioni et al., 2008) and the preference of the field has shifted toward biological replicates over technical replicates just as array based transcriptomics evolved earlier (Fang and Cui, 2011). Biological replication is important as without estimating the variability within a group it is not possible to estimate true differences between the groups under observation and conclusions from such results cannot be generalized (Auer and Doerge, 2010; Robasky et al., 2014). In transcriptomic studies one aims for an adequate trade-off between the number of replicates as well as reads such that it is cost-effective and provides sufficient statistical confidence for interpretation (Fang and Cui, 2011; Liu et al., 2014; Todd et al., 2016).

### Recommended number of replicates and reads

Higher numbers of biological replicates provide a better representation of biological variance across samples for transcriptomic analysis (Churchill, 2002; Yang and Speed, 2002; Fang and Cui, 2011). It also reduces chances of any “bad replicates” skewing results or adding unwanted variation (Gierlinski et al., 2015). However, it is not practical nor is it always possible to have very large number of replicates for each condition in biological studies due to time, sample availability and cost constraints and the addition of replicates beyond a certain level has diminishing returns and will depend on the nature of the study's goal (Liu et al., 2014). Our results are similar to previous studies that show a greater proportional increase in the number of differentially expressed genes when moving from two to three replicates compared to from three to four. Similar trends of decreasing DE genes were observed when replicates and reads were removed from analysis. Having read numbers in the same range across samples is a consideration. Similarly, higher genome coverages with large number of reads have also been shown to result in higher DE gene detections but within limits (Haas et al., 2012). It has been reported that with reads below 5 M a considerable drop in DE gene numbers is observed, but increasing reads beyond 10 M results in only slight increments in detection and adding replicates at this point has a much higher effect instead (Haas et al., 2012; Liu et al., 2014). Our analyses with sets of randomly sampled 5–75% of the total available reads while maintaining four replicates depicted a similar trend in consensus with previous findings. In addition, here we performed a combined analysis with reduced reads (25% subset) and reducing replicate numbers (Marguerat and Bahler, 2010; Martin and Wang, 2011; Williams et al., 2014). The results depicted that the set of three replicates and 25% reads (~7.5–13 M) detected a similar number of DE genes as four replicates and 25% reads with most of the genes commonly detected between the two sets. Moreover, the number of DE genes detected with three and four replicates with 100% reads was not much greater than when 25% reads with the most similar numbers were used. Thus, based on all the above it is recommended that designing an experiment with as low as three good replicates (high correlation coefficients, indicating reproducibility across samples) and at least 5–10 million reads per sample would be the most efficient and cost effective design for a microbial transcriptomics study.

It is not just the “number” of DE genes detected with increasing number of replicates and reads, but also whether the genes detected with a set of fewer replicates and/or reads forms a subset of the genes detected with the set of higher number of replicates and reads or if they are “newly” identified genes. We performed an overlap assessment for the DE genes detected from all combinations of analysis to look at core as well as exclusive subsets of genes and used RT-qPCR for validating a representative few. The major reasons for exclusively detected genes were related to FDR and *p*-value cut-offs and filtering out of the lowly expressed genes via the independent filtering in DESeq2. Genes with expression and *p*-values very close to the cut-off range moved above and below the limits with changes in the numbers of reads and replicates. Due to these reasons such genes varied between data subsets and were therefore not found among the commonly detected core genes. Thus, by applying well considered cut-offs tailored to the aim of the study as well as the expected gene expression profile one can potentially avoid missing out on genes relevant to the analyses. This signifies the importance of choosing appropriate threshold limits set for each study and examining the broader context of expression changes by considering pathway and operons as two examples. Deeper sequencing coverage (more replicates and reads) may still be required for studies that are more discovery-based than hypothesis-driven (Yang et al., 2012; Wilson et al., 2013). A higher coverage may also be desirable to deal with sequencing errors and polymorphisms larger than single base differences (Wang et al., 2009; Williams et al., 2014) or to detect a rare transcript or variant or lowly expressed genes.

### Differentially expressed genes and experimental design considerations

The majority of typical laboratory experiments would control for much of the variation identified in this study by using media prepared from all the same stock, by using defined media, and/or by randomizing treatment/control samples if they had to be cultured on different dates. However, there may be circumstances such as long term continuous growth studies where rich media cannot be prepared from the same lot or batch, in which case being able to build such variables into statistical, or other, tests is an important consideration. RNA-seq analysis identified significantly differentially expressed genes based on medium lot and culture date differences, two major variation sources in the dataset. The highly differentially expressed iron acquisition and metabolism genes, were a likely consequence of differing amounts of iron in the two media lots. Indeed, in this study RNA-seq was a tool for predictive biology since we hypothesized and confirmed the two LB medium lots had different iron contents. The large (~two-fold) difference in measured iron contents was surprising and may be of broader interest to the research communities that use this media. Our results are in agreement with earlier studies showing the importance of culture medium choices in transcriptomics (Blair et al., 2013). In this study, other experimental factors beyond the type of media used were shown to be important.

### Significance of the available data set

This dataset has relevance to researchers interested in *Bacillus* biology. The genus *Bacillus* contains representatives such *B. thuringiensis* (e.g., BMB171, Bt407)*, Bacillus subtilis* (e.g., *BSn5*), *Bacillus anthracis* (e.g., Ames), and others (Aronson et al., 1986; Alam et al., 2011; Bishop and Robinson, 2014) that occupy diverse ecological niches and have important biotechnological roles. Moreover, this is a rich dataset with four biological replicates and high genome coverage (85–465X), which may interest researchers in testing, developing, and evaluating bioinformatics software for RNA-seq analyses in future for example in testing/developing normalization algorithms and mapping tools etc. (Peixoto et al., 2015; Seyednasrollah et al., 2015; Medina et al., 2016). This data set could also be utilized toward generating and testing new globally acceptable RNA-seq analysis pipelines such as the recently developed “PANDORA” (Moulos and Hatzis, 2015), which could then permit further comparisons and developments and improving existing RNA-seq analysis pipelines.

In conclusion this study outlines the significance of a well-controlled experimental design, choice of threshold parameters, adequate number of reads and replicates toward an efficient and cost effective transcriptomics study. Moreover, the depth and complexity of this RNA-seq data will be useful to others for a range of studies such as for insights into *Bacillus* physiology and for further developments in the field of bioinformatics for microbial transcriptomics.

## Author contributions

LH, DP, and SB planned and initiated the study. TL grew the cultures and harvested samples for transcriptomics. DK generated RNA-seq data and assisted with RT-qPCR experiments. PM generated and analyzed RT-qPCR data. TM conducted elemental analyses. PM, CW, LH, and SB analyzed the data and wrote the manuscript. All authors edited the manuscript and approved the final manuscript.

## Funding

This work is sponsored by the Laboratory Directed Research and Development Program of Oak Ridge National Laboratory and used laboratories supported by the BioEnergy Science Center (BESC). BESC is a US Department of Energy Bioenergy Research Center supported by the Office of Biological and Environmental Research in the DOE Office of Science. This manuscript has been authored by UT-Battelle, LLC under Contract no. DE-AC05-00OR22725 with the US Department of Energy. The United States Government retains and the publisher, by accepting the article for publication, acknowledges that the United States Government retains a non-exclusive, paid-up, irrevocable, world-wide license to publish or reproduce the published form of this manuscript, or allow others to do so, for United States Government purposes. The Department of Energy will provide public access to these results of federally sponsored research in accordance with the DOE Public Access Plan (http://energy.gov/downloads/doe-public-access-plan). The funders had no role in study design, data collection and analysis, decision to publish, or preparation of the manuscript.

### Conflict of interest statement

The authors declare that the research was conducted in the absence of any commercial or financial relationships that could be construed as a potential conflict of interest.

## Supplementary material

A summary is provided in Data Sheet 1.
