# Supplementary material for: Replicates, Read Numbers, and Other Important Experimental Design Considerations for Microbial RNA-seq Identified Using Bacillus thuringiensis Datasets
Source: Front Microbiol. 2016 May 31;7:794. doi: 10.3389/fmicb.2016.00794 (PMC4886094; doi:10.3389/fmicb.2016.00794)
Supplement: Supplementary file 1 [file DataSheet1.docx]

**Summary of Supplementary Files**

**Data Sheet 1:** Summary of supplementary files. This file.

**Data Sheet 2:** Growth data, sampling details and sequence read mapping results.

**Data Sheet 3:** DESeq2 input data files (ATCC10792 & CT43).

**Data Sheet 4:** DESeq2 result files (ATCC10792 & CT43): medium effect and CT43 DE iron genes.

**Data Sheet 5:** DESeq2 result files (ATCC10792): culture date effect.

**Data Sheet 6:** DESeq2 result files (CT43): culture date effect.

**Data Sheet 7: Supp. Table 1:** Primer sequences for RT-qPCR; **Supp. Figure 1 & 2:** Multivariate linear correlation analysis of variance within biological replicates.

**Data Sheet 8:** Replicates (2-4): Input files & results + 2-4 replicates and 25% (~7.5-13 M) read combination input and result files.

**Data Sheet 9:** Reads (5%-75%): Input files & results.
