# Supplementary material for: Replicates, Read Numbers, and Other Important Experimental Design Considerations for Microbial RNA-seq Identified Using Bacillus thuringiensis Datasets
Source: Front Microbiol. 2016 May 31;7:794. doi: 10.3389/fmicb.2016.00794 (PMC4886094; doi:10.3389/fmicb.2016.00794)
Supplement: Supplementary file 7 [file DataSheet7.docx]

**Data Sheet 7.**

**Supp. Table 1: List of RT-qPCR primers used for validation of RNA seq results for medium lots and culture dates.**

| **Sequence Description** | **Primer Sequence** |
| --- | --- |
| **Medium effect validation genes** |  |
| bthur0008_13460_FP | TCGTTGAGGGAGGAAAAGAA |
| bthur0008_13460_RP | CGCACCTGAAGATCGGAATA |
| bthur0008_6770_FP | AGGCGCTGTTAATACGATGC |
| bthur0008_6770_RP | CTCCAACTCCGCCATACACT |
| bthur0008_28360_FP | ACAGCTTGGTTTTTCGGAGA |
| bthur0008_28360_RP | CCCATACTCCGACATGCATAC |
| bthur0008_34530_FP | AGGGATTGCAATTGGATCAG |
| bthur0008_34530_RP | TATGCTTCCAGACACCCACA |
| bthur0008_41720_FP | GTGCTGATGTAGCGAAGCAA |
| bthur0008_41720_RP | GTGGACCTCTTCCGAACTCA |
| bthur0008_34520_FP | TACAACTGGTAGCCCGGAAG |
| bthur0008_34520_RP | GCCCCGTTATACGGATCAAT |
| bthur0008_38600_FP | TGCGGCATTAACAGAGGTGT |
| bthur0008_38600_RP | ACTTTCCCAAGCACAAGAGC |
| **Date effect validation genes** |  |
| bthur0008_61310_FP | TCATGCACAGGTGAACGTCG |
| bthur0008_61310_RP | ACGGGTAATTTCTCCAACCCC |
| bthur0008_38200_FP | CCAACGTGTTGCGTTAGGTA |
| bthur0008_38200_RP | GCATTGCAACACGGAGTTTA |
| bthur0008_3370_FP | ATAATCCGAAGCGTGATTGG |
| bthur0008_3370_RP | ATCATATTCCCATGCCGAAC |
| bthur0008_4210_FP | ACAAGTTGGCCCTGAGTACG |
| bthur0008_4210_RP | TAGACCCGCTAACCATTCCA |
| bthur0008_52220_FP | GTGGCTATGGATGGCTTGTT |
| bthur0008_52220_RP | AAGGGGATTTTCCCTTCTTG |
| bthur0008_17630_FP | TGCGATTGCAAACTTTATGG |
| bthur0008_17360_RP | CCCAAGTAACGCAAATGGAT |


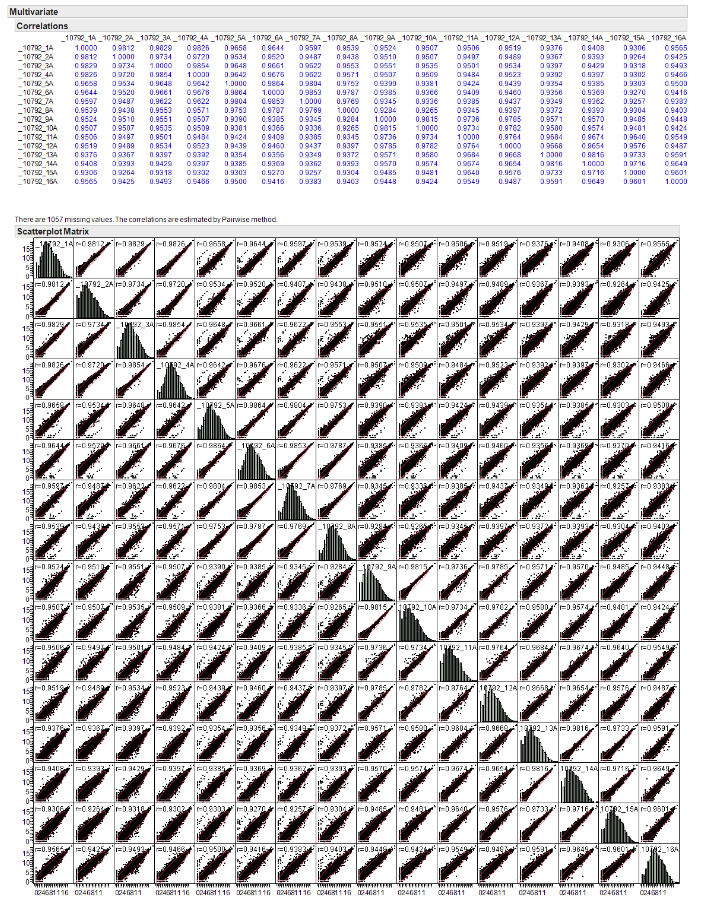


**Supp. Figure 1: Multivariate correlation analysis summarizing variation among biological replicates of strain ATCC10792.**


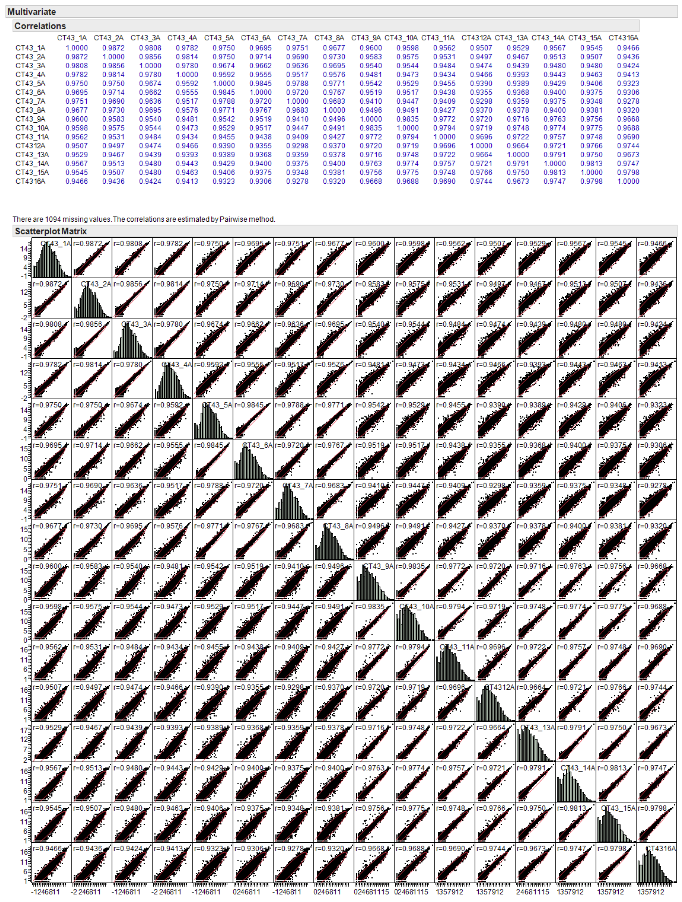


**Supp. Figure 2: Multivariate correlation analysis summarizing variation among biological replicates of strain CT43**
